# Development of a Novel Electrochemical Biosensor Based on Carbon Nanofibers–Gold Nanoparticles–Tyrosinase for the Detection of Ferulic Acid in Cosmetics

**DOI:** 10.3390/s20236724

**Published:** 2020-11-24

**Authors:** Alexandra Virginia Bounegru, Constantin Apetrei

**Affiliations:** Department of Chemistry, Physics and Environment, Faculty of Sciences and Environment, “Dunărea de Jos” University of Galaţi, 47 Domnească Street, 800008 Galaţi, Romania; alexandra.meresescu@ugal.ro

**Keywords:** ferulic acid, cyclic voltammetry, tyrosinase, biosensor, cosmetic products

## Abstract

The present paper deals with the electrochemical behavior of three types of sensors based on modified screen-printed electrodes (SPEs): a sensor based on carbon nanofibers (CNF/SPE), a sensor based on nanofibers of carbon modified with gold nanoparticles (CNF-GNP/SPE) and a biosensor based on nanofibers of carbon modified with gold nanoparticles and tyrosinase (CNF-GNP-Ty/SPE). To prepare the biosensor, the tyrosinase (Ty) was immobilized on the surface of the electrode already modified with carbon nanofibers and gold nanoparticles, by the drop-and-dry technique. The electrochemical properties of the three electrodes were studied by cyclic voltammetry in electroactive solutions, and the position and shape of the active redox peaks are according to the nature of the materials modifying the electrodes. In the case of ferulic acid, a series of characteristic peaks were observed, the processes being more intense for the biosensor, with the higher sensitivity and selectivity being due to the immobilization of tyrosinase, a specific enzyme for phenolic compounds. The calibration curve was subsequently created using CNF-GNP-Ty/SPE in ferulic acid solutions of various concentrations in the range 0.1–129.6 μM. This new biosensor allowed low values of the detection threshold and quantification limit, 2.89 × 10^−9^ mol·L^−1^ and 9.64 × 10^−9^ mol·L^−1^, respectively, which shows that the electroanalytical method is feasible for quantifying ferulic acid in real samples. The ferulic acid was quantitatively determined in three cosmetic products by means of the CNF-GNP-Ty/SPE biosensor. The results obtained were validated by means of the spectrometric method in the infrared range, the differences between the values of the ferulic acid concentrations obtained by the two methods being under 5%.

## 1. Introduction

Ferulic acid (3-metoxy-4-hydroxycinnamic acid) (FA) is found in fruit, juices and packaged fruit, packaged fruit juices, vegetables, rice, oats, alcoholic beverages and many Chinese medicinal plants, like *Angelica sinensis*, *Cimicifuga heracleifolia*, etc. [1,2]. FA is known as an antiaging [3], anti-inflammatory [4], antithrombotic [5], antidiabetic [6], antiulcerigen [7], antihemolitic [8], antiviral [9] and even anti-carcinogenic agent [10]. Due to the antibacterial effect, it may also be used in certain implants [11]. Ferulic acid plays an important role in the human body and is absorbed in the small intestine as a major metabolite of chlorogenic acids [12,13]. FA is known for its powerful antioxidant effect, being useful in the elimination of free radicals [14]. Additionally, it has a good pain-killing effect, and is suggested as an aid in treating neuropathic pain [13,15].

Considerable research has focused on the study of the antioxidant properties of ferulic acid and its use in food industry, pharmacy and cosmetology [16,17,18]. Preventing skin ageing is one of the main issues in contemporary cosmetology and aesthetic medicine. Antioxidant compounds with demonstrated efficacy include ferulic acid. Originally, FA was used in cosmetic products as a stabilizer of other popular antioxidants, like vitamin C and vitamin E [19]. However, research shows that this compound may be an active ingredient supporting the intracellular anti-antioxidant defense systems [4]. That is why ferulic acid is used in antiaging cosmetic formulations [20,21]. Due to its ability to inhibit the main enzyme of melanogenesis, it is used in anti-spot cosmetic formulations [22].

Ferulic acid is used in manufacturing face masks, as well as antioxidant, protective and hydrating creams/lotions. The concentration of ferulic acid recommended in cosmetic products of this type is between 0.5 to 1%. Ferulic acid is also used in cosmetology and cosmetic surgery. It is often used at a concentration of 12% and in combination with vitamin C and hyaluronic acid [22]. There is research stating that ferulic acid, according to the concentration, may be efficient in dermatological issues, such as atopic dermatitis [23]. Therefore, determining the concentration of ferulic acid is important in the food industry and cosmetic industry.

At present, there are several quantitative methods to determine FA, such as high-performance liquid chromatography, gas chromatography, thin layer chromatography, spectrophotometry, chemoluminescence, micellar electrokinetic chromatography, etc. [24,25,26]. However, these traditional methods require longer working time and more complex devices and procedures [27,28]. In the past few years, electrochemical analysis has proved promising in application prospects in various fields, like quality control in the food industry, pharmaceutical industry and cosmetic industry, due to high sensitivity, low detection threshold and accessible working stages [29,30].

The electrochemical behavior and the quantitative determination of FA have been researched using several working electrodes, modified with various nanomaterials, like reduced graphene oxide [2], graphene functionalized with poly(diallyldimethylammonium chloride [31], multi-wall carbon nanotubes and silver nanoparticles [1] or a nanocomposite consisting of MgO and single-wall carbon nanotubes (MgO/SWCNTs), to which 1-Butyl-3-methylimidazolium-bis- (trifluoromethylsulfonyl)-imide was added [29]. The performance of the modified electrodes depends on the properties of the modifier, which affect the selectivity and sensitivity of these electrodes in FA detection.

In the analysis of antioxidant compounds, research has focused on developing electrochemical biosensors, mainly those based on tyrosinase, due to their low costs, quick response and low energy consumption [32,33]. Tyrosinase is a monooxygenase monophenol that catalyzes the oxidation of the phenol group to o-quinone, which is the commonest enzyme used in the electrochemical determination of phenolic compounds [34,35,36,37]. As electrochemical transducers for the determination of phenolic compounds by means of tyrosine-based sensors, several types of electrodes were used, such as Sonogel–carbon [38], glassy carbon electrodes [34] and screen-printed electrodes (SPEs) [39].

Immobilizing enzymes on nanomaterial-modified screen-printed electrodes makes it possible for nanomaterials’ properties and advantages to act synergically [37,40,41]. Nanomaterials are suitable as electrochemical sublayers, having good catalytic activity, a stable surface for enzyme immobilization and excellent conductivity [42]. The following is a presentation of the most recent sensors developed for the detection of ferulic acid, the electrochemical technique used, the linearity domain and the value of the detection threshold (Table 1).

As seen in Table 1, the specialized literature contains few studies on biosensors aimed at the detection of ferulic acid. As a result, devising new biosensors for FA detection is important from a theoretical and practical point of view.

The purpose of the present paper is to study the electrochemical behavior and the qualitative and quantitative determination of ferulic acid by means of three types of electrodes: a screen-printed electrode based on carbon nanofibers (CNF/SPE), a screen-printed electrode based on nanofibers of carbon modified with gold nanoparticles (CNF-GNP/SPE) and a biosensor obtained by modifying CNF-GNP/SPE by immobilizing tyrosinase on its surface. The novelty of such biosensors consists in use of two classes of nanomaterials, carbon nanofibers and gold nanoparticles, and an enzyme for the sensitive and selective detection of ferulic acid from real samples. The synergic effect of such sensitive materials could be exploited to develop highly sensitive and selective biosensors. Additionally, the electroanalytical method is validated for the quantification of ferulic acid in various cosmetic products by means of a classic method, infrared spectrometry.

## 2. Materials and Methods

### 2.1. Reagents and Solution

The screen-printed electrode based on carbon nanofibers (CNF/SPE) and the one based on carbon nanofibers and gold nanoparticles (CNF-GNP/SPE) were purchased from Metrohm DropSens (Oviedo, Spain). CNF-GNP/SPE was used for the subsequent modification with tyrosinase (Ty).

Potassium ferrocyanide, sodium diphosphate, potassium bromide and phosphoric acid were bought from Sigma-Aldrich (St. Louis, Missouri, SUA). The preliminary tests made use of phosphate buffer solution 10^−1^ M (PBS) pH 7.0 and a solution of potassium ferrocyanide 10^−3^ M–PBS 10^−1^ M.

The phosphate buffer solution 10^−1^ M (PBS) pH 7.0 was used as a support electrolyte for all electrochemical measurements. PBS was prepared with NaH_2_PO_4_ and Na_2_HPO_4_ (Sigma-Aldrich, St. Louis, MO, USA). After calculating and weighing the amounts, they were dissolved in ultrapure water (obtained by means of a Milli-Q Millipore system, Bedford, MA, SUA). The pH of the PBS was checked and corrected by means of a pH-meter (WTW, Weilheim, Germany).

The ferulic acid (analytical purity) used in electroanalytical studies was purchased from Sigma-Aldrich. To prepare the stock solution of ferulic acid (10^−3^ M), the accurate amount of ferulic acid was dissolved in the PBS solution at pH 7.0. The tyrosinase enzyme (EC 232-653-4, from mushroom), with a concentration of 5370 U/mg was purchased from Sigma-Aldrich (St. Louis, MO, USA). To immobilize the enzyme, a tyrosinase solution was used at a concentration of 50 μg·µL^−1^ dissolved in a phosphate buffer solution (0.01 M, pH 7.0).

### 2.2. Apparatus and Electrodes

The voltammetric measurements were performed by means of an EG&G potentiostat/galvanostat (Princeton Applied Research, Oak Ridge, TN, USA), the 263A model, controlled by ECHEM software. An electrochemical cell (Princeton Applied Research, Oak Ridge, TN, USA) was used, with a volume of 50 mL. The reference electrode used was Ag/AgCl, and the auxiliary electrode was a platinum wire. These materials are suitable for electrochemical tests in an aqueous environment, being stable and toxicity free, and the working electrode was in turn CNF/SPE, CNF-GNP/SPE and the CNF-GNP-Ty/SPE biosensor. To dissolve substances and homogenize solutions, an Elmasonic ultrasonic bath (Carl Roth GmbH, Karlsruhe, Germany) was used. The measurement and adjustment of the pH was performed by Inolab pH 7310 (WTW, Weilheim, Germany).

### 2.3. Biosensor Preparation

To prepare the biosensor, the tyrosinase enzyme was immobilized on the surface of the CNF-GNP/SPE electrode by the dripping technique, followed by reticulation by means of glutaraldehyde. In order to perform this procedure, a tyrosinase enzyme solution was prepared in PBS 0.01 M (pH 7.0) with a concentration of 50 μg·μL^−1^. Ten microliters of solution were added on the surface of the CNF-GNP/SPE working electrode. After the solvent evaporated, the biosensor was placed above a recipient of glutaraldehyde 2%, for 1 min. Thus, the glutaraldehyde vapors triggered enzyme reticulation. Then the biosensor was kept at room temperature to dry, for 5 min, in a desiccant. The CNF-GNP-Ty/SPE biosensor was kept at a temperature of 4 °C in a desiccant until it was used [49].

### 2.4. Cosmetic Product Analysis

The cosmetic products chosen for analysis were purchased from specialized stores, based on their composition, as reported by the manufacturer. They have different presentation forms, i.e., a serum, an emulsion and a cream.

The first cosmetic product tested was the serum called The Ordinary Resveratrol 3% + Ferulic Acid 3%. This formula combines high concentrations of two of the strongest skin antioxidants: resveratrol and ferulic acid, and also contains propanediol in the composition.

Sabio, a toning cream for eyes and lips with ferulic acid and *Aloe vera*, is a cosmetic product rich in nutritive, antioxidant, hydrating and remineralising substances. In its composition, there are the following components: water, *Aloe vera* juice, rice oil, macadamia oil, avocado oil, cucumber extract, glycerin, croton lechery resin extract, cetearyl olivate, sorbitan olivate/vegetable emulsifier, sodium levulinate, sodium anisate, vitamin E, ferulic acid, cedar wood essential oil, juniper, lemongrass, geranium and melissa.

The make-up removing emulsion Detox Aslavital Mineralactiv is aimed at the efficient removal of make-up and skin cleaning and it has remineralizing and detoxifying effects. The emulsion contains a purified marine glycogen Cobiodefender EMR, which, in association with 100% natural clay, grants the skin the necessary energy to reduce the effects of urban pollution, electronic pollution and UV radiation. Among the active ingredients, the manufacturer confirms the presence of ferulic acid, without mentioning its concentration.

For the electrochemical analysis of the cosmetic products, the following procedure was used. Different amounts of cosmetic products were weighed and dissolved in 50 mL phosphate-buffered solution 10^−1^ M pH 7.0. Three replicates for each cosmetic compound were prepared for each sample and concentration. The analyses of cosmetic products were carried out by using cyclic voltammetry in the potential range between −0.4 V and 1.3 V at a scan rate of 0.1 V·s^−1^.

The infrared spectra of cosmetics and FA reference samples were acquired with a Bruker ALPHA FT-IR spectrophotometer (BrukerOptik GmbH, Ettlingen, Germany) in the range of 4000–500 cm^−1^. The FT-IR spectrophotometer includes an attenuated total reflectance (ATR) sampling module. The ZnSe crystal was carefully rinsed with isopropanol between measurements in order to remove the impurities. All spectra were registered with a resolution of 4 cm^−1^ and 32 scans per sample vs. background (dry empty ATR crystal) using OPUS software (BrukerOptik GmbH, Ettlingen, Germany).

## 3. Results and Discussion

### 3.1. The Voltametric Behavior of Electrodes in PBS and Potassium Ferro-Cyanide

Preliminary analysis evaluated the electrochemical behavior of CNF/SPE, CNF-GNP/SPE and CNF-GNP-Ty/SPE. The electrolytic solutions used were as follows: phosphate-buffered solution (PBS pH 7.0) and potassium ferrocyanide solution 10^−3^ M–PBS 10^−1^ M.

The first potential interval used was −1.0 V and +1.3 V, in which the signal was unstable for both electrodes. So, the potential of the negative vertex was gradually increased, until a stable signal was obtained, in the cases of the PBS solution, the potassium ferrocyanide-PBS and ferulic acid-PBS. A stable signal was obtained for all the solutions under study within the potential range between −0.4 and +1.3 V.

This potential range was initially used for the study of the electrochemical behavior of CNF/SPE, CNF-GNP/SPE and CNF-GNP-Ty/SPE immersed in PBS 10^−1^ M (pH 7.0). The scan rate was 0.1 V·s^−1^. A cathodic peak was observed, in the case of CNF-GNP/SPE and CNF-GNP-Ty/SPE at E = 0.440 V (current −13.361 μA) and E = 0.455 V (current −11.286 μA), respectively. These peaks occur due to the modification of the screen-printed electrodes with gold nanoparticles and tyrosinase, respectively, in the case of the biosensor. The background current was reduced for all the three electrodes used.

The next test included the immersion of each of the three electrodes in a solution of potassium ferrocyanide 10^−3^ M-PBS 10^−1^ M (pH 7.0). The potential ranged between −0.4 and +1.3 V, and the scan rate was 0.1 V·s^−1^. In the cyclic voltammograms obtained with the three electrodes, we may observe an anodic and a cathodic peak. These peaks are due to the oxido-reduction process of the ferrocyanide occurring on the electrode surface. The characteristics of the peak pair observed when immersing CNF/SPE, CNF-GNP/SPE and CNF-GNP-Ty/SPE in potassium ferrocyanide solution are shown in Table 2.

As shown in Table 2, E_1/2_ has close values for CNF/SPE and CNF-GNP/SPE and a lower value for CNF-GNP-Ty/SPE. All three electrodes have an I_pc_/I_pa_ ratio above 1. Taking into account the I_pc_/I_pa_ and ΔE values, which are higher than the theoretical values, it may be considered that the redox processes are quasi-reversible [50]. The highest peaks are seen for CNF-GNP/SPE, which shows higher sensitivity in detecting potassium ferrocyanide and the synergic effect of gold nanoparticles in electrodetection. The presence of Ty on the biosensor surface is clearly evinced by the differences seen between the electrochemical behavior of CNF-GNP/SPE and CNF-GNP-Ty/SPE.

### 3.2. Electrochemically Active Surface Area

**Figure 1:**
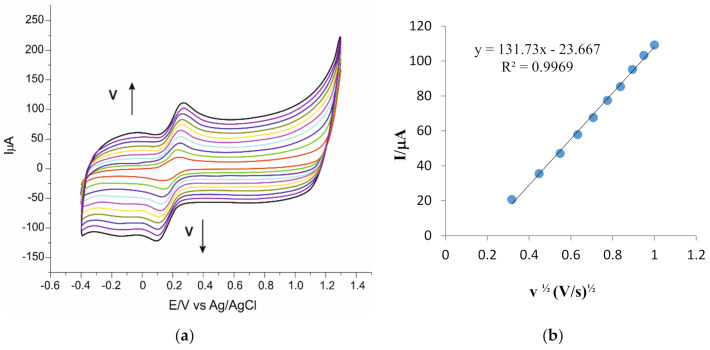

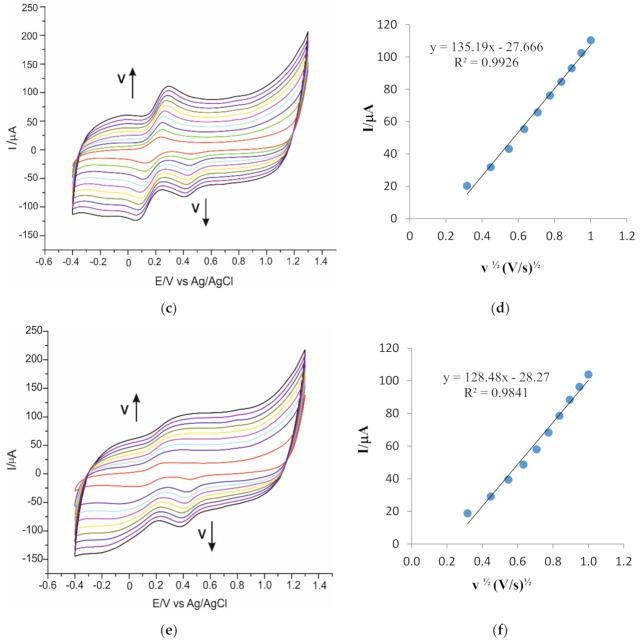
Cyclic voltammograms of CNF/SPE (**a**), CNF-GNP/SPE (**c**), CNF-GNP-Ty/SPE (**e**) immersed in 10^−3^ M K_4_[Fe(CN)_6_]—10^−1^ M KCl solution registered with scan rates in the range 0.1–1.0 V·s^−1^. The cyclic voltammograms with different colors correspond to different scan rates. Plot of Ipc vs. v^1/2^ for CNF/SPE (**b**), CNF-GNP/SPE (**d**), CNF-GNP-Ty/SPE (**f**).

The next step was the recording of the cyclic voltammograms at various scan rates (0.1–1.0 V·s^−1^), using the potassium ferrocyanide solution 10^−3^ M–10^−1^ M PBS (pH 7.0). Figure 1a,c,e show that the intensity of the peaks corresponding to the processes of oxido-reduction of the ferrocyanide increases with the scan rate.

For all electrodes, it can be seen that there is a linear dependence between the current of the anodic peak and the square root of the scan rate (Figure 1b,d,f), which proves that the electrochemical process is controlled by the diffusion of the electroactive species [51]. To calculate the active area of the electrodes, the Randles–Ševčík equation was used [52].

$$I_{\mathrm{pa}}=268,600\cdot n^{3/2}\cdot A\cdot D^{1/2}\cdot C\cdot v^{1/2}$$

where: I_pa_ is the anodic current (A), n is the number of electrons transferred in the redox process, A is the area of the active surface of the electrode (cm^2^), D is the diffusion coefficient (cm^2^·s^−1^), C is the concentration (mol·cm^−3^) and v is the scan rate (V·s^−1^).

Taking into account the diffusion coefficient of the ferrocyanide ion D = 7.26 × 10^−6^ cm^2^·s^−1^ [53] and the equation of the linear regression I_pa_ vs. v^1/2^, the equation yields the value of the active area surface for CNF/SPE, CNF-GNP/SPE and CNF-GNP-Ty/SPE. The results are included in Table 3.

The CNF-GNP-Ty/SPE biosensor has the lowest value of the active surface, as the Ty immobilized on the electrode surface does not take part in the process of oxido-reduction of the ferrocyanide, proving selectivity. The two screen-printed sensors have an almost equal active surface, but modifying the electrode surface with gold nanoparticles accounts for the larger active area of CNF-GNP/SPE. The results agree with the intensities of the anodic peaks seen in Figure 1a,c,e. Carbon nanofibers are useful in electrode modification due to their remarkable properties (good mechanic and thermal conductivity, large surface, surface-to-volume ratio, low ohmic resistance) resulting in a faster electron transfer. Besides, gold nanoparticles were used to develop sensors and biosensors, as they have good conductibility and improved electrocatalytical capacity, are biocompatible and modifying the electrode is quite easy [33,54,55]. The Ty enzyme provides detection selectivity in multicomponent solutions [49].

### 3.3. The Voltammetric Responses of Electrodes in Ferulic Acid Solution

**Figure 2:**
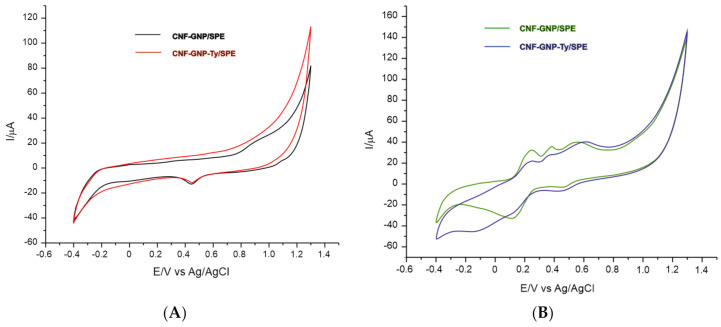
(**A**) Cyclic voltammograms of the CNF-GNP/SPE (black line) and the CNF-GNP-Ty/SPE (red line) in 10^−1^ M PBS solution (pH 7.0). (**B**) Cyclic voltammograms of the CNF-GNP/SPE (green line) and the CNF-GNP-Ty/SPE (blue line) in 10^−3^ M ferulic acid–10^−1^ M PBS solution (pH 7.0). Scan rate: 0.1 V·s^−1^.

In detecting ferulic acid at a higher sensitivity and selectivity, the CNF-GNP/SPE and CNF-GNP-Ty/SPE electrodes were used. Figure 2 shows the cyclic voltammograms of the CNF-GNP/SPE and CNF-GNP-Ty/SPE electrodes immersed in (A) 10^−1^ M PBS (pH 7.0) and (B) 10^−3^ M ferulic acid solution–10^−1^ M PBS (pH 7.0).

Both cases evince three anodic peaks and two cathodic peaks of different intensity and potential, related to the oxidation and the reduction, respectively, of the ferulic acid at the surface of the sensitive element. This electrochemical behavior is similar to that observed in other previous studies [56]. Table 4 shows the results obtained for the analysis of the redox peak pair (I) seen in cyclic voltammograms.

In the case of CNF-GNP-Ty/SPE, the potential of cathodic peak I is lower, and this displacement towards negative values of the potential shows that the reduction process is strongly influenced by the presence of the enzyme [57,58]. This detection at a lower potential shows that the reduction process needs a lower activation energy in the biosensor case, it being a property of the enzymes [59]. So, the biosensor has superior sensitivity and selectivity as compared to the sensor in detecting ferulic acid. In regard to the interaction manner between the electrode and the ferulic acid, the specialized literature contains several studies on the mechanism of electrochemical detection of the ferulic acid by means of the electrochemical sensors [56].

Some authors described just one oxidation peak attributed to an irreversible process involving the transfer of an electron and a proton, resulting in the formation of the phenoxy radical, which upon dimerization forms a polymeric film on the electrode surface [2].

Other researchers noticed two anodic oxidation peaks of FA [60,61], or even three anodic peaks [62] and had different explanations for the mechanism of formation. One study proposed a mechanism of FA electrooxidation involving the formation of intermediaries of caffeic acid and metoxyhydroquinone, the final product of the oxidation being 5-hydroxyferulic acid (5-HFA). According to this mechanism, the first oxidation peak is due to the oxidation of the FA molecules in solution, and the second anodic peak was attributed to the oxidation of the FA molecules adsorbed on the electrode surface [61]. Manaia et al. [60] consider that the first stage of irreversible oxidation of FA, corresponding to the first peak, is due to the formation of a diphenol, and the second peak occurs due to the oxidation of the double bond in the side chain of FA. Both studies admitted that ferulic acid has a higher redox potential than caffeic acid, as mentioned in several papers [56,63].

**Figure 3:**
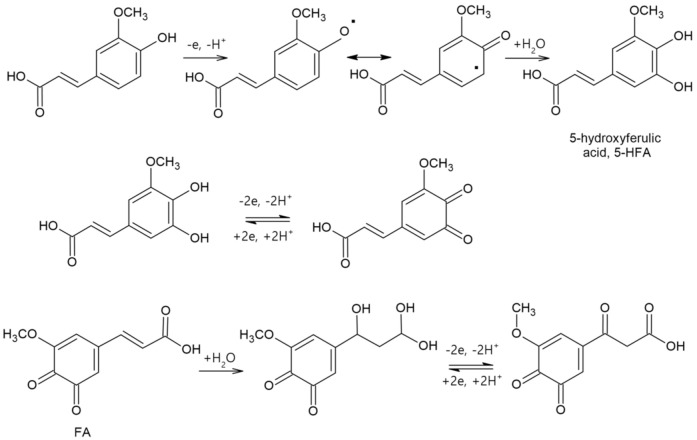
Proposed mechanism for the electrochemical oxidation/reduction of ferulic acid (FA) [56].

The mechanism of the electrooxidation and electroreduction reactions of FA in the case of the sensor and the biosensor devised in this study is shown in Figure 3.

The reduction processes are those of the quinonic products formed by anodic oxidation, which occur in two stages, resulting in two clearly defined cathodic peaks.

The enzyme immobilization was confirmed by voltammetric analysis, as seen in Figure 3. It was found that the enzyme immobilized in the biosensor catalyzes the hydroxylation reactions of the benzene nucleus and the oxidation reactions of the *ortho*-diphenolic derivative to the corresponding quinone [64]. That is why reduction peak I is substantially modified, being the main difference between the sensor and the biosensor. Obtaining lower values of the peak potential suggests a rapid electron transfer process in the redox process of ferulic acid at the level of the active surface [43]. Upon analyzing the signal obtained with CNF-GNP-Ty/SPE, the potential of cathodic peak I has a much lower value than in the case of CNF-GNP/SPE, which means that the reduction process of the product of electrochemical oxidation of ferulic acid requires a lower activation energy in the case of the biosensor [1]. In addition, the value of the cathodic current of CNF-GNP-Ty/SPE is higher than in the case of CNF-GNP/SPE, proving that the biosensor is more sensitive to the electrochemical detection of the oxidation product of ferulic acid. Additionally, the I_pc_/I_pa_ ratio is higher for the biosensor. This increase in the cathodic current in the case of the biosensor is due to tyrosinase, which catalyzes the oxidation of ferulic acid [65].

As a result, in the case of CNF-GNP-Ty/SPE, the oxidation of the ferulic acid occurs by a mechanism involving the transfer of two electrons and two protons [62]. As a result of the ferulic acid oxidation, the main product obtained is the o-quinonic derivative of ferulic acid [62]. The tyrosinase immobilized on the sensor surface increases the biosensor selectivity, which is confirmed mainly by the increase in the cathodic peak current and the movement of the cathodic peak at a more negative potential as compared to the potential observed for CNF-GNP/SPE.

**Figure 4:**
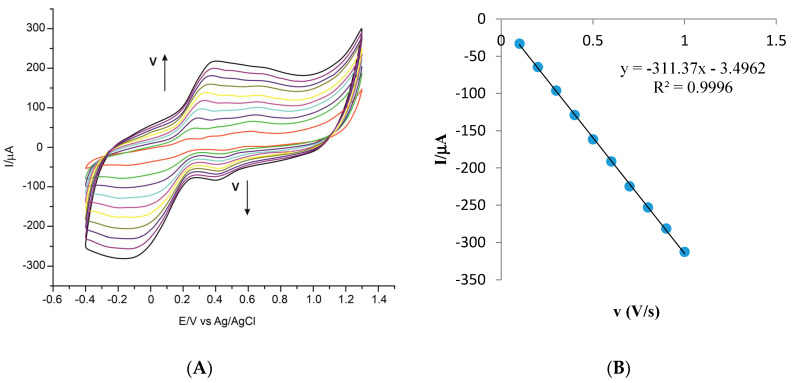
(**A**) Cyclic voltammograms of CNF-GNP-Ty/SPE in 10^−3^ M ferulic acid–10^−1^ M PBS solution (pH 7.0). The cyclic voltammograms with different colors correspond to different scan rates. (**B**) The plot of cathodic peak currents (i_c_) vs. scan rates.

The next stage meant the study of the electrochemical behavior of the two electrodes for scanning at various rates (within the range 0.1 V·s^−1^ and 1.0 V·s^−1^), each time increasing the scan rate by 0.1 V·s^−1^, in ferulic acid solution 10^−3^ M (the electrolyte support was 10^−1^ M PBS of pH 7.0). Figure 4A shows the cyclic voltammograms obtained for CNF-GNP-Ty/SPE with different scan rates and Figure 4B shows the linear dependence between the currents of the cathodic peak and the scan rates.

The linear dependence between cathodic peak current (i_c_) and v confirms that the redox process of ferulic acid is controlled by adsorption [66]. As a result, the reduction process is governed by the Laviron equation [67].

$$i_{c}=\frac{n^{2}F^{2}\Gamma \mathrm{Av}}{4\mathrm{RT}},$$

where: n—number of electrons involved in the redox process; F—Faraday constant; Γ—surface concentration of the electroactive species; A—area of electrode; v—scan rate; R—universal constant of gases; T—absolute temperature.

Comparing the results obtained with CNF-GNP-Ty/SPE and CNF-GNP/SPE, it can be stated that in both cases the reduction process is controlled by the adsorption, the process being faster in the case of the biosensor (as seen when comparing the slopes of the two equations of linear adjustment in Table 5). By means of the Laviron equation, the values of the surface concentration of the electroactive species (Γ) were calculated from the slope of the linear equation between i_c_ and v, and the results are included in Table 5.

The values of Γ are similar to those obtained for other biosensors based on tyrosinase used in detecting phenolic compounds [68,69].

These results lead to the assertion that the biosensor has better electroanalytical properties in detecting ferulic acid. Besides, the presence of tyrosinase provides superior selectivity to the biosensor in complex samples. The immobilization of tyrosinase, together with carbon nanofibers and gold nanoparticles, leads to better biosensitivity and conductivity, these nanomaterials having a synergic effect in biodetection. Hence, the quantitative tests made use of the biosensor manufactured in this study.

### 3.4. Effect of Ferulic Acid Concentration on the Biosensor Response

**Figure 5:**
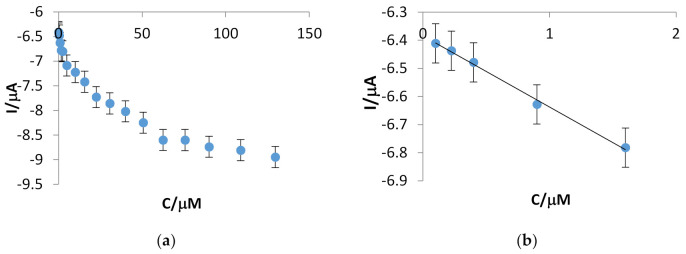
The calibration curve of the biosensor in the concentration ranges 0.1–129.6 μM (**a**) and 0.1–1.6 μM (**b**).

Subsequently, cyclic voltammetry was used to detect ferulic acid in various concentrations by means of CNF-GNP-Ty/SPE. It may be observed that the intensity of reduction peak I increases with the concentration of the ferulic acid within the concentration range studied, from 0.1 to 129.6 μM (Figure 5).

The increase in the reduction current is linear with concentration within the range between 0.1 and 1.6 μM, and the linear regression equation is y = −0.2529x − 6.3845 (R^2^ = 0.9961, n = 5), with a limit of detection (LOD) of 2.89 × 10^−9^ mol·L^−1^ and a limit of quantification (LOQ) of 9.64 × 10^−9^ mol·L^−1^. The LOD of the CNF-GNP/SPE sensor in the detection of FA was also determined. The value is 4.32 × 10^−7^ mol·L^−1^, a value two orders of magnitude lower than the value obtained in the case of the biosensor.

When comparing the analytical parameters, such as the linearity interval and LOD, it was noted that the biosensor has better performances than most (bio)sensors included in Table 1. These results are due to nanomaterials and the enzyme in the sensitive element of the biosensor and the favorable interaction with the ferulic acid.

### 3.5. Stability, Reproducibility of Fabrication, Repeatability, Interference Studies

The biosensor is stable and may be used for more than 50 measurements by cyclic voltammetry in FA-containing solutions. In regard to the reproducibility of the manufacturing method, no differences larger than 2% were obtained between identically prepared biosensors immersed in FA solutions of the same concentration. Similarly, the variation of the biosensor response to FA determination in solutions of the same concentration, when taking it out of the solution, rinsing and repeating the cyclic voltammogram, was no higher than 3%. The biosensor displayed very good selectivity, the potential and the current of the cathodic peak remaining virtually the same for added compounds in the cosmetic products, like propandiol, glycerine, vitamin E, etc.

### 3.6. FA Measurement in Cosmetic Products

**Figure 6:**
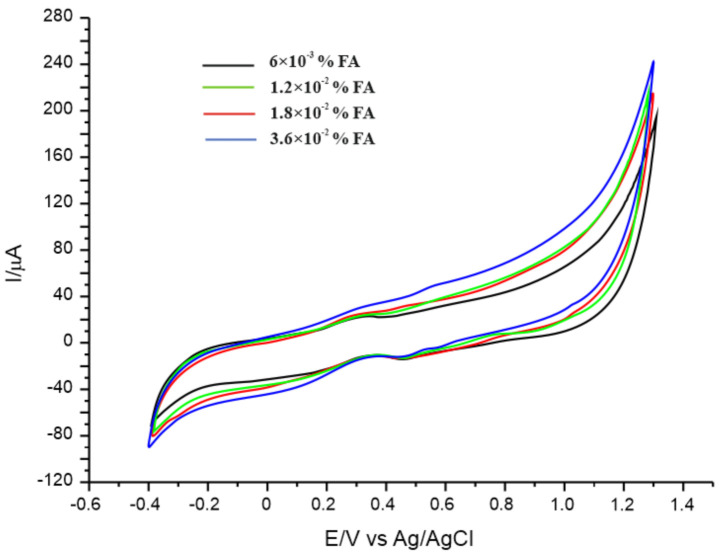
Cyclic voltammograms of CNF-GNP-Ty/SPE immersed in serum antioxidant Ordinary solutions of different concentrations: 6 × 10^−3^% FA (black line); 1.2 × 10^−2^% FA (green line), 1.8 × 10^−2^% FA (red line), 3.6 × 10^−2^% FA (blue line). Scan rate was 0.1 V·s^−1^. The cyclic voltammogram of blank solution (10^−1^ M PBS, pH 7.0) was subtracted from the cyclic voltammograms obtained in cosmetic samples and PBS.

To check the practicability and feasibility of the method proposed, CNF-GNP-Ty/SPE was used to detect ferulic acid in cosmetic products with various presentation forms and consistency: serum, cream and emulsion. The cyclic voltammograms performed for all these cosmetic products show the peaks due to ferulic acid, and the currents increase with the concentration of cosmetic product. Figure 6 shows the cyclic voltammograms of CNF-GNP-Ty/SPE immersed in solutions of Ordinary antioxidant serum of various concentrations. The representative peaks may be seen for the electrochemical processes of the ferulic acid, and reduction peak I was used for quantification.

Taking into account the current of the cathodic peak, the amount of cosmetic product tested and the equation of the calibration line, the ferulic acid concentrations were calculated for the cosmetic products under investigation, obtaining the results included in Table 6. To validate the voltammetric method, the cosmetic products were also analyzed by the infrared range spectrometric method. The amounts of ferulic acid in the cosmetic products were calculated from the calibration equation corresponding to the peak at 1050 cm^−1^, related to the stretch vibration of the C-O phenol group [70]. All experiments were carried out in triplicate, and the results can be seen in Table 6. In the case of FTIR analysis, the standard samples were obtained by mixing the pure ferulic acid with solid potassium bromide at different levels of percentage concentration, between 0.1 and 5%. The absorbance of the samples at 1050 cm^−1^ was quantified. By plotting the absorbance as a function of concentration, the calibration line was developed. The absorbance of the cosmetics was quantified directly and, by interpolation in the calibration line, the FA concentration was calculated.

It can be observed that the FA concentrations in the cosmetic products obtained by the two methods are close in value, which proves that the method using the CNF-GNP-Ty/SPE biosensor is useful for FA quantification with sufficient accuracy. In the case of the Ordinary product, for which the manufacturer indicates 3% FA concentration, it can be seen that the results are close to the indicated ones, thus proving the accuracy of the two methods in FA detection.

## 4. Conclusions

A novel (bio)sensor was designed and manufactured, based on nanomaterials and tyrosinase for the electrochemical detection of ferulic acid. The CNF-GNP-Ty/SPE biosensor proved to be useful in the analysis of ferulic acid in cosmetic products. Quantification based on the cathodic peak allowed the selective detection of FA in complex matrices. The use of cyclic voltammetry as a detection method allowed the study of FA detection, reaching excellent analytical performance applicable in electroanalysis. The FA concentrations obtained by means of the CNF-GNP-Ty/SPE biosensor are very close to the results obtained by the standard FTIR method or the values indicated by the manufacturer. The method proposed in the present study has a series of advantages, such as good accuracy, simplicity and low cost. Besides, the method is very accurate and is also versatile, so it can be used in routine analysis in the quality control of cosmetic products, pharmaceuticals, food supplements and other types of samples. The method based on the biosensor could also be feasible for the analysis of ferulic acid in food products.

## Figures and Tables

**Table 1:** Detection technique, linear range, sensitivity and limit of detection (LOD) of some voltammetric sensors and biosensors used for ferulic acid detection.

(Bio)Sensor	Detection Technique	Linear Range	LOD	Reference
multi-walled carbon nanotube decorated with silver nano-particle-modified carbon paste electrodes (CPE/MWCNTs-Ag)	CV	4.0 × 10^−8^–1.0 × 10^−3^ M	3.0 × 10^−8^ M	[1]
MgO/SWCNTs-1-Butyl-3-methylimidazolium bis(trifluoromethylsulfonyl)imide paste electrode MgO/SWCNTs-[B*mim*][Tf2N]-CPE	DPV	0.009–450 µM	3.0 nM	[29]
carbon nanotubes decorated with manganese dioxide nanoparticles f-MWCNTs/MnO_2_ modified GCE	SWV	0.082–220 μM	10 nM	[43]
polypyrrole-multi-walled carbon nanotube-modified glassy carbon electrode PPy-MWCNTs/GCE	CV SWV	3.32 × 10^−6^–2.59 × 10^−5^ M	1.17 × 10^−6^ M	[44]
graphene oxide sheets (GOs) and multi-walled carbon nanotube (MWCNT) nanocomposites modified glassy carbon electrode GOs/MWCNTs/GCE	CV	0.24 μM–32 μM 88 μM–1.23 mM	0.08 μM	[25]
TiO_2_ nanoparticle-decorated, chemically reduced graphene oxide-modified glassy carbon electroder GO-TiO_2_-GCE	CV DPV	0.1 μM–1 μM 1 μM–300 μM	0.01 μM	[45]
a glassy carbon electrode modified with functional multi-walled carbon nanotubes that are decorated with MnO_2_ nanoparticles	CV	0.082–220 μM	10 nM	[46]
droplet-based electrochemical sensor	CV	0.0257–0.515 mM	0.024 mM	[47]
oxygen electrode-based *Trametes versicolor laccase* ferrocene-modified screen-printed graphite electrode-based *Trametes versicolor* laccase	HV	0.05–0.2 μM 2.0–10.0 μM	0.01 μM 1.8 μM	[48]

CV—cyclic voltammetry; DPV—differential pulse voltammetry; SWV—square wave voltammetry; HV—hydrodynamic voltammetry; SWCNT—single walled carbon nanotubes; GCE—glassy carbon electrode.

**Table 2:** Electrochemical parameters obtained from voltammograms of sensors immersed in K_4_[Fe(CN)_6_] solution 10^−3^ M–10^−1^ M phosphate-buffered solution (PBS) (pH 7.0).

Electrode	E_pa_^1^ (V)	E_pc_^2^ (V)	E_1/2_^3^ (V)	ΔE ^4^ (V)	I_pa_^5^ (µA)	I_pc_^6^ (µA)	I_pc_/I_pa_
CNF/SPE	0.227	0.149	0.188	0.078	20.3664	−21.6371	1.04541
CNF-GNP/SPE	0.231	0.146	0.188	0.084	20.6964	−21.7651	1.05164
CNF-GNP-Ty/SPE	0.242	0.089	0.165	0.152	18.8795	−21.575	1.1428

^1^ Potential of the anodic peak; ^2^ potential of the cathodic peak; ^3^ half wave potential; ^4^ ΔE = E_pa_ − E_pc_; ^5^ current of the anodic peak; ^6^ current of the cathodic peak; CNF/SPE—carbon nanofibers/screen-printed electrode; CNF-GNP/SPE—carbon nanofibers—gold nanoparticles/screen-printed electrode; CNF-GNP-Ty/SPE—carbon nanofibers—gold nanoparticles—tyrosinase/screen-printed electrode.

**Table 3:** The area of the active surface of the electrodes used in the analysis.

Electrode	Active Area (cm^2^)
CNF/SPE	0.1819 ± 0.0036
CNF-GNP/SPE	0.1868 ± 0.0037
CNF-GNP-Ty/SPE	0.1774 ± 0.0035

**Table 4:** The values of the parameters obtained from the cyclic voltammograms of all the electrodes immersed in 10^−3^ M ferulic acid solution (the electrolyte support was 10^−1^ M PBS of pH 7.0).

Electrode	E_pa_^1^ (V)	E_pc_^2^ (V)	E_1/2_^3^ (V)	I_pa_^4^ (µA)	I_pc_^5^ (µA)	I_pc_/I_pa_
CNF-GNP/SPE	0.240	0.124	0.182	32.813	−33.108	1.008
CNF-GNP-Ty/SPE	0.229	−0.123	0.176	21.721	−46.096	2.122

^1^ Potential of the anodic peak; ^2^ potential of the cathodic peak; ^3^ half wave potential; ^4^ current of the anodic peak; ^5^ current of the cathodic peak.

**Table 5:** The linear fitting equations (i_c_ vs. v), R^2^ and Γ.

Electrode	Equation	R^2^	Γ (mol·cm^−2^)
CNF-GNP/SPE	y = −2.585 × 10^−5^ x − 2.348 × 10^−5^	0.9994	5.02 × 10^−11^
CNF-GNP-Ty/SPE	y = −3.1137 × 10^−5^ x − 3.4962 × 10^−5^	0.9996	6.05 × 10^−11^

y = I_pc_; x = *v*.

**Table 6:** The results of FA quantification in cosmetics.

Cosmetic Product	c% FA FTIR Method	c% FA Biosensor Method
Ordinary	2.932	3.114
Gerovital	0.090	0.104
Sabio	0.096	0.112

c%—percentage concentration; Relative standard deviation (RSD) = 2%.
